# Structures and Dynamics of Native-State Transmembrane Protein Targets and Bound Lipids

**DOI:** 10.3390/membranes11060451

**Published:** 2021-06-17

**Authors:** Michael Overduin, Catharine Trieber, R. Scott Prosser, Louis-Philippe Picard, Joey G. Sheff

**Affiliations:** 1 Department of Biochemistry, University of Alberta, Edmonton, AB T6G 2H7, Canada; ctrieber@ualberta.ca; 2 Department of Chemistry, University of Toronto, UTM, Mississauga, ON L5L 1C6, Canada; scott.prosser@utoronto.ca (R.S.P.); louisphilippe.picard@utoronto.ca (L.-P.P.); 3 Human Health Therapeutics Research Centre, National Research Council Canada, Ottawa, ON K1A 0R6, Canada; Joey.Sheff@nrc-cnrc.gc.ca

**Keywords:** drug discovery, GPCR, ion channel, native nanodisc, phosphoinositide, transmembrane protein, transporter

## Abstract

Membrane proteins work within asymmetric bilayers of lipid molecules that are critical for their biological structures, dynamics and interactions. These properties are lost when detergents dislodge lipids, ligands and subunits, but are maintained in native nanodiscs formed using styrene maleic acid (SMA) and diisobutylene maleic acid (DIBMA) copolymers. These amphipathic polymers allow extraction of multicomponent complexes of post-translationally modified membrane-bound proteins directly from organ homogenates or membranes from diverse types of cells and organelles. Here, we review the structures and mechanisms of transmembrane targets and their interactions with lipids including phosphoinositides (PIs), as resolved using nanodisc systems and methods including cryo-electron microscopy (cryo-EM) and X-ray diffraction (XRD). We focus on therapeutic targets including several G protein-coupled receptors (GPCRs), as well as ion channels and transporters that are driving the development of next-generation native nanodiscs. The design of new synthetic polymers and complementary biophysical tools bodes well for the future of drug discovery and structural biology of native membrane:protein assemblies (memteins).

## 1. Introduction

Obtaining accurate kinetic and structural information about molecular interactions of biologically relevant states of therapeutic targets is the foundation of modern drug discovery. However, this endeavour is complicated by the fact that most targets are embedded in asymmetric lipid bilayers with signaling lipids, post-translational modifications, cofactors and heteromeric subunits. The native assemblies are essentially impossible to reconstitute using recombinant methods and detergents, which strip off the lipids that are bound in vivo. Overcoming this challenge represents a key goal of an open innovation community known as the styrene maleic acid lipid particle (SMALP) network. Our challenge is to progress from in vitro to ex vivo structural biology, allowing currently inaccessible molecular machines to be excised from endogenous sources and purified intact for structure–function analysis. Targeting native states while keeping the original lipids, cofactors, modifications and partner proteins bound is clearly critical for discovery of drug molecules with higher selectivity and fewer side effects.

**Figure 1:**
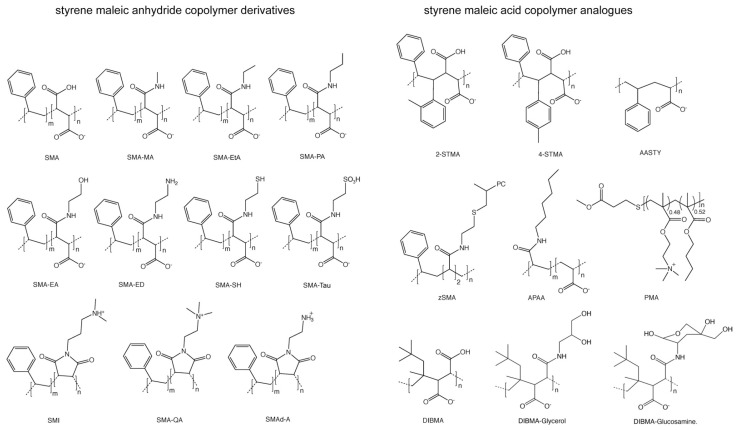
Types of copolymers used to solubilize transmembrane proteins within native nanodiscs. The hydrolyzed derivatives of styrene maleic anhydride include styrene-*co*-maleic acid (SMA) [4], styrene-*alt*-maleamic acid (SMA-MA) [5], SMA ethanolamine (SMA-EA) [6], SMA ethylenediamine (SMA-ED) [7], SMA with sulfhydrils (SMA-SH), [8], SMA with taurine (SMA-Tau) [9], styrene-co-maleimide (SMI) [10], styrene maleimide quaternary ammonium (SMA-QA) [11], and dehydrated SMA ethylenediamine copolymer (SMAd-A). The analogues of SMA include poly(2-methylstilbene-alt-maleic anhydride) (2-STMA), poly(4-methylstilbene-alt-maleic anhydride) (4-STMA) [7,12], acrylic acid-*co*-styrene (AASTY) [13], zwitterionic SMA (zSMA) [14], alkyl polyacrylic acid (APAA) [15], polymethacrylate (PMA) [16], and diisobutylene-*alt*-maleic acid (DIBMA) [17] as well as its glycerol- and glucosamine-containing derivatives.

**Figure 2:**
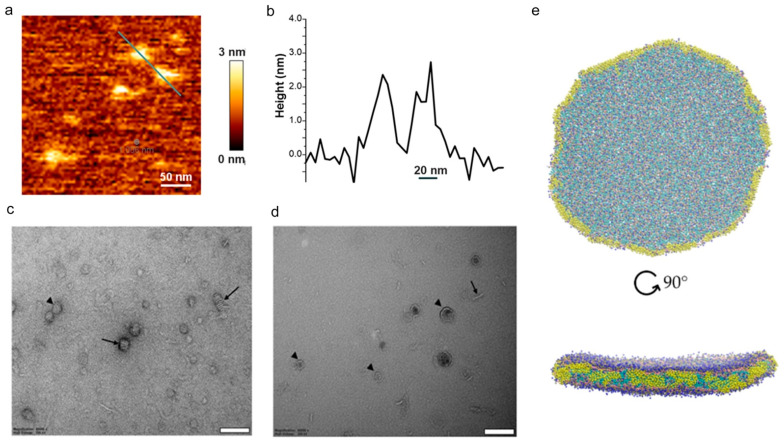
Structures of nanodiscs made with synthetic copolymers. (**a**) AFM image of nanodiscs formed from a 1:1 ratio of DIBMA and dimyristoyl phosphatidylcholine (DMPC) lipid, with a green line indicating the position of the height profile including a pair of nanodiscs in (**b**). Micrographs of these nanodiscs collected by negative-stain TEM (**c**) and cryo-TEM (**d**), showing face-on (arrowheads) and edge-on (arrows) particles. Scale bar 100 nm. (**e**) Coarse-grained molecular dynamics simulations of 40 nm nanodiscs from top and side views. Taken from [18].

This effort has led to the development of a growing set of synthetic SMA-derived copolymers including SMA and its derivatives (Figure 1) that convert membranes from cells or tissues into native nanodiscs containing membrane:protein assemblies (memteins) for high-throughput screening and structure determination. The outcomes to date consist of the detergent-free purification of a growing number of membrane-embedded proteins including GPCRs of the rhodopsin-like class A type, bacteriorhodopsins, and multisubunit complexes such as ion channels, and transporters. The structures of nanodiscs themselves can be visualized and modelled by a variety of computational and biophysical methods including atomic force microscopy (AFM) and transmission electron microscopy (TEM), as shown in Figure 2.

Seminal studies highlighting the potential to characterize native-state complexes within SMA nanodiscs are detailed in Table 1. Structures of transmembrane assemblies in nanodiscs are emerging, leading to a wave of new information about how signaling lipids such as phosphoinositides (PIs) modulate receptor function and stabilize multimeric states of receptors such as Eph2 [1]. The signaling interactions of PI lipids with soluble proteins through the FYVE (Fab-1, YGL023, Vps27, and EEA1), PH (pleckstrin homology) and PX (phox homology) domains is better understood and has led to the discovery of the PI code [2,3]. The centrality of this code in eukaryotic cell organization, signal transduction and membrane trafficking suggests that common features and principles may emerge with transmembrane proteins. In this review, the recent progress in the field is considered with a focus on transmembrane protein families of interest to the pharmaceutical industry as well as strategies to overcome remaining challenges.

## 2. Native GPCR–Lipid Complexes

Understanding native GPCR function is fundamentally important to the pharmaceutical industry. These receptors bind more therapeutic agents than any other family, and are targeted by approximately one-third of all marketed drugs [19]. Genomic studies suggest that there are over 800 human GPCRs [20], of which less than 15% have been subjected to large-scale drug discovery screens [21,22]. GPCRs generally respond to a diversity of signals and mediate cellular responses through the interactions of heterotrimeric G proteins, GPCR kinases and arrestins, and are particularly sensitive to their lipid membrane environment [23,24,25]. Their pharmacology is also complicated by the fact that many GPCRs signal through multiple G proteins and non-cognate proteins, often via unique oligomerization states [26]. This complex signal response can be biased by specific orthosteric and allosteric ligands and thus represents an active area of research [27,28,29,30,31]. The first structure of a GPCR appeared in 2000 [32], and has been followed by hundreds of further structures due to advances in the development of recombinant protein fusions, thermostabilisation strategies, lipidic cubic phase (LCP) and cryo-EM systems [33]. However, many challenges remain, underlining the potential for further growth. Post-translational modifications are important for GPCR expression and function [34] yet are rarely retained, and neither are the biological lipids, multimeric assemblies or protein sequences found in vivo. Further, there is evidence that solubilization with detergents must be carefully optimized to minimize disruptions to native-state structural dynamics [35]. This infers an ongoing need for efficient optimization and preparation of native source complexes suitable for analysis of the signaling activities and structures, particularly of states most relevant to drug discovery.

The first GPCR to be solubilized without detergents using the SMALP system is the human adenosine A2A receptor (A_2A_R), as expressed both in *Pichia (P.) pastoris* and human embryonic kidney (HEK) cells [36]. After the GPCR assembly was released into nanodiscs using SMA(2:1) copolymer (~2% *w/v*) and purified using N-terminal His_10_ tags, it displayed ligand binding activity similar to that of the protein in yeast membranes, and exhibited a stable helical structure that withstood repeated freeze–thaw and storage cycles. The change of positions of Trp residues from accessible to buried positions as ligands bind to wild-type and mutant A_2A_R can be monitored by fluorescence spectroscopy [37]. Attachment of fluorescent reporters to Cys residues provides additional probes for monitoring ligand-dependent conformational changes, while associated lipids including PE and PC can be identified by tandem mass spectrometry. Single-molecule sensitivity can be achieved with fluorescence correlation spectroscopy to characterize ligand binding by this receptor in SMALPs [38].

Dopamine receptors are GPCRs that contain a cysteine-rich extracellular and a long intracellular C-terminus, and can form functional heterodimers with adenosine receptors [39]. The dopamine D1 receptor is expressed at low levels in HEK cells and has been purified using SMA(3:1) copolymer. Once stably integrated in native nanodiscs, this GPCR is helical based on circular dichroism (CD) spectra, and binds the SCH23390 antagonist and neurotensin peptide based on radioligand and microscale thermophoresis (MST) assays [40]. The dopamine and ghrelin receptors form heteromultimeric complexes in hypothalamic neurons. Their tetrameric assembly can be purified using SMA (2:1) and is competent for ligand binding, recruiting a pair of G protein trimers [41]. The ghrelin receptor (GHS-R1a) and melatonin receptor (MT1R) can be solubilized into 13 nm discs from liposomes and *Pichia pastoris* membranes using SMA(2:1) or SMA(3:1). With the yeast preparations, a polymer concentration of 2.5% *w/v*, low salt and low temperature are optimal for retaining MT1R stability and for binding G protein and arrestin [42]. Another class A GPCR, the cannabinoid receptor 1 (CB1), was solubilized from insect cells using SMA2000 and purified to yield 15 nm discs that bind a conformationally specific antibody [43]. The protein is more thermostable in discs than in N-dodecyl-β-maltoside (DDM) detergent and can be used for fluorescence-activated cell sorting and surface plasmon resonance (SPR) experiments, providing avenues to screen against antigen or compound libraries. Together, this suggests that there is broad utility of such polymers for structural analysis and high-throughput screening of diverse GPCR targets in native nanodiscs.

**Figure 3:**
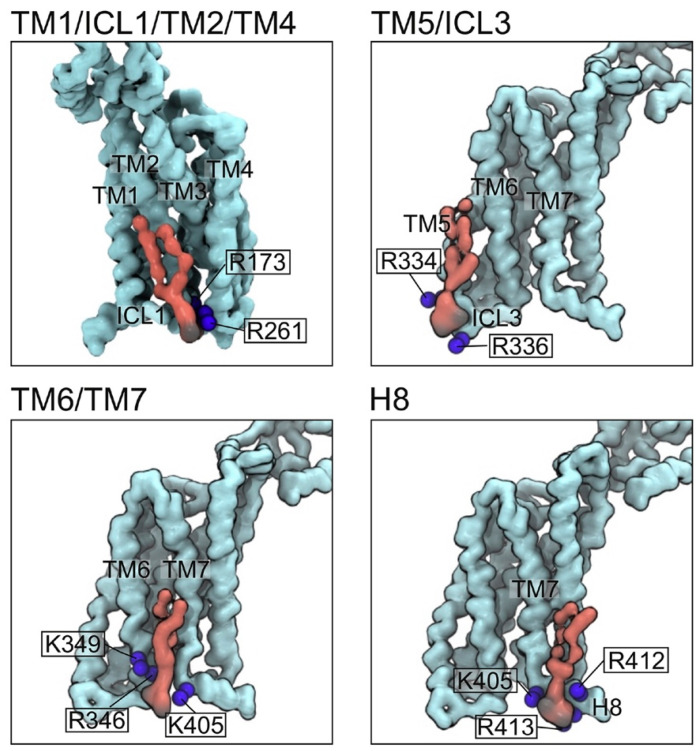
Phosphoinositide interactions with a model of the glucagon receptor. Cross-grain molecular dynamics simulation of this class B GPCR (light blue) in an asymmetric lipid bilayer reveals a variety of binding poses of PI(4,5)P_2_ molecules (red) in the inner leaflet, showing the juxtaposition of phosphate groups (black) and Arg and Lys residues (blue spheres). Taken from [49].

The stability, activity and selectivity of GPCRs depends on their interactions with lipids. The array of lipids complexed with the β1 adrenergic receptor (β1AR), β2AR and neurotensin receptor 1 (NTSR1) include cholesterol, phosphatidylserine, phosphoinositide and phosphatidic acid molecules based on tandem mass spectrometry analysis of their collisional dissociation [24,44,45,46]. These receptors as well as A_2A_R bind most tightly to PtdIns(4,5)P_2_, which serves to increase G protein coupling and GTPase activity. An exogenous PtdIns(4,5)P_2_ molecule is seen to bridge the complexed structure of NTSR1 and β-arrestin, stabilizing their active conformation [47] and suggesting that such ternary states are viable targets. The interactions of lipids including gangliosides in the outer leaflet and PIs in the inner leaflet can be modelled and reveal multiple potential binding sites in GPCRs of either class A [48] or class B [49], illustrating such multivalent interactions (Figure 3). NTSR1 complexes have been expressed in insect cells and solubilized using polymethacrylate (PMA) co-polymer, yielding nanodiscs with higher levels of stimulation of both G_q_ and G_i1_ heterotrimers than detergent micelle preparations [50]. PMA offers different sidechains and greater polycation compatibility than SMA, although the yields and purities are lower than those achieved with detergents.

A variety of additional copolymers are being developed to purify such transmembrane targets and investigate biological lipid and ligand interactions. This has been demonstrated with the A_2A_R and human V_1a_ vasopressin receptor (V_1a_R), as expressed in transfected HEK cells [10]. They were both solubilized using cationic styrene maleimide (SMI) copolymer, which offers broad pH, Ca^2+^ and Mg^2+^ tolerance as well as milder solubilization activity, and yields slightly smaller particles with diameters from 6–12 nm. While the most widely used SMA(2:1) copolymer generally offers the highest membrane solubilization efficiency, its activity is compromised at low pH or high polycation levels where the polymer can precipitate. The related DIBMA copolymer has been used to solubilize the β2-adrenoceptor (β2AR), which is sensitive to phospholipid composition [51]. Comparison of binding activities of β2AR in membrane or DIBMA-based nanodiscs reveals comparable interactions with full agonist (isoprenaline), antagonist (propranolol) and inverse agonist (ICI 118,551), while DDM detergent significantly destabilizes the protein [52]. Given such options, synthetic copolymer kits have been developed by Cube Biotech and Orbiscope for comparing the performance of various copolymers when purifying active target proteins.

## 3. Microbial Rhodopsins in Native Nanodiscs

Bacteriorhodopsins form a large superfamily of seven transmembrane helix (7TM) proteins that all contain a covalently bound retinal molecule. When this chromophore absorbs light, it undergoes *cis–trans* isomerization, inducing a conformational change in the protein. In particular, light converts the inverse agonist-bound off position to a full agonist state. The latter state catalyzes the replacement of GDP by GTP on transducin, which is an associated heterotrimeric G protein that triggers a response in rod cells of the retina. These 7TM bacterial proteins are topologically similar to mammalian class A GPCRs, which bind diffusible agonists and exhibit analogous lipid-dependent dynamics and conformational changes that also transduce allosteric information through G proteins.

**Figure 4:**
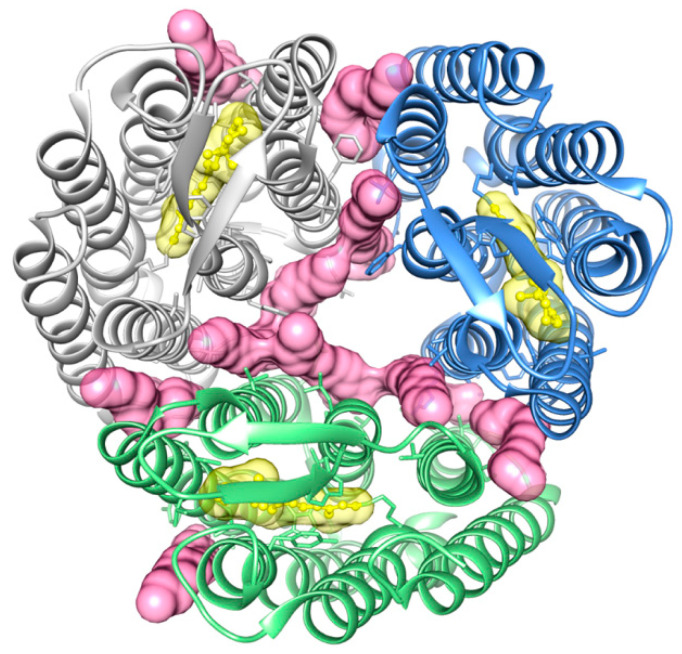
Structure of trimeric *Haloquadratum walsbyi* rhodopsin [59]. The bound monoolein lipids (pink) are evident between the sets of seven transmembrane helices of each subunit, which are colored white, blue and green, while retinol is yellow. The structure is from PDB ID: 5ITC, and the image is from [69].

The first atomic resolution structure of a lipid-bound protein determined using SMALPs appeared in 2017. The crystal structure of a bacteriorhodopsin trimer from *Haloquadratum walsbyi* was resolved to 2.0 Å following its purification with a C-terminal double His_6_ tag in the presence of 2.5% (*w/v*) SMA(3:1) copolymer. The protein had been transferred from *Escherichia (E.) coli* membranes which were spiked with 1.5% (*w/v*) DMPC to increase the yield relative to levels achievable with DDM detergent. This allowed gentle release of the memteins into a lipidic cubic phase (LCP) system for *in meso* crystallization. The resulting structure reveals the orientation of a set of interacting monoolein molecules as well as the bound *trans*-retinal, with SMALPs offering an improvement in resolution [59] (Figure 4). Endogenous lipids were not bound tightly enough to be visible in the structure. Nonetheless, their interactions could be modelled from the monoolein lipids and suggest key roles in trimer assembly, flexibility and the photocycle.

A recent study compared thermally stable and unstable rhodopsins from *Rubrobacter xylanophilus* (RxR) and *Halobacterium salinarum* sensory rhodopsin I (HsSRI). Both were produced in *E. coli* with C-terminal His_6_ tags and then solubilized in SMA(2:1) copolymer [58]. A high copolymer concentration of 5% (*w/v*) was used to increase protein yield without needing to add exogenous DMPC. Four sets of trimers of RxR are evident by dynamic light scattering analysis of the 54 nm diameter nanodiscs, which are five times larger than typically seen for most SMALPs. Average particle diameters of 30 nm were observed by AFM, with nanodiscs stacked in sets of one, two or three. Approximately 700 lipid molecules were present per nanodisc along with the four trimers. This corresponds to an average ratio of 57 lipids for every protein molecule, which is higher than earlier estimates of lipids associated with the *E. coli* PagP β barrel in SMALPs, potentially indicating greater membrane retention or a more ordered lipid microenvironment. The time-course of rhodopsin photocycles in SMALPs was very similar to that measured in egg PC liposomes but differs from that in DDM detergent preparations, which hinder proton pumping activity. The more unstable HsSRI protein is deactivated by DDM but retains its color and activity when solubilized and purified in SMALPs. The SMA–HsSRI complex binds NaCl with a dissociation of 34 mM, and this interaction influences its absorption of light, indicating that SMALPs provide a more native-like membrane environment.

The endogenous lipid interactions of archeorhodopsin and bacteriorhodopsin, which are lost with detergent, are maintained in SMALPs. Native mass spectrometry revealed that these receptors complexed with diphytanyl glycerol lipid ligands (S-DGD and 2DP) in native membranes isolated using SMA(3:1) copolymer and DMPC [56]. This study compared n-octyl-β-d-glucoside detergent preparations and membrane scaffold protein (MSP)-based nanodiscs and showed that SMALPs are uniquely suited to solubilizing and stabilizing the correctly folded form of the protein while retaining bound biological lipids, which have long been known to preserve its stability and activity [70].

Another study focused on the sensory rhodopsin II of *Natronomonas pharaonis* (NpSRII), which mediates photorepellent responses to light. This receptor and its transducer protein were purified from *E. coli* membranes using single C-terminal His_6_ tags and 2.5% *w/v* SMA(2:1) copolymer [60]. The photocycle reaction times of the complex reconstituted into SMALPs from *E. coli* membranes are similar to those from DDM and liposome preparations. However, the decay of the M photointermediate is accelerated in SMALP preparations. This can be attributed to high local proton concentrations as a result of the carboxylic groups on the SMA molecules. In contrast, this decay was decelerated in membranes which have altered lipid compositions and lower charge densities, as this reduces reprotonation rates for key intermediates in the reaction pathway. Hence, both lipid and polymer charge can affect signaling, providing an impetus for designing uncharged or zwitterionic copolymers to retain endogenous activity. This protein has also been solubilized into larger nanodiscs (up to 35 nm) using DIBMA copolymer [18]. The ratio of lipid to protein influences the photocycle of the rhodopsin, which also increases lipid dynamics, indicating the importance of optimizing the concentrations and ratios of polymers.

## 4. Cryo-Electron Microscopy of Multisubunit Complexes in Native Nanodiscs

**Figure 5:**
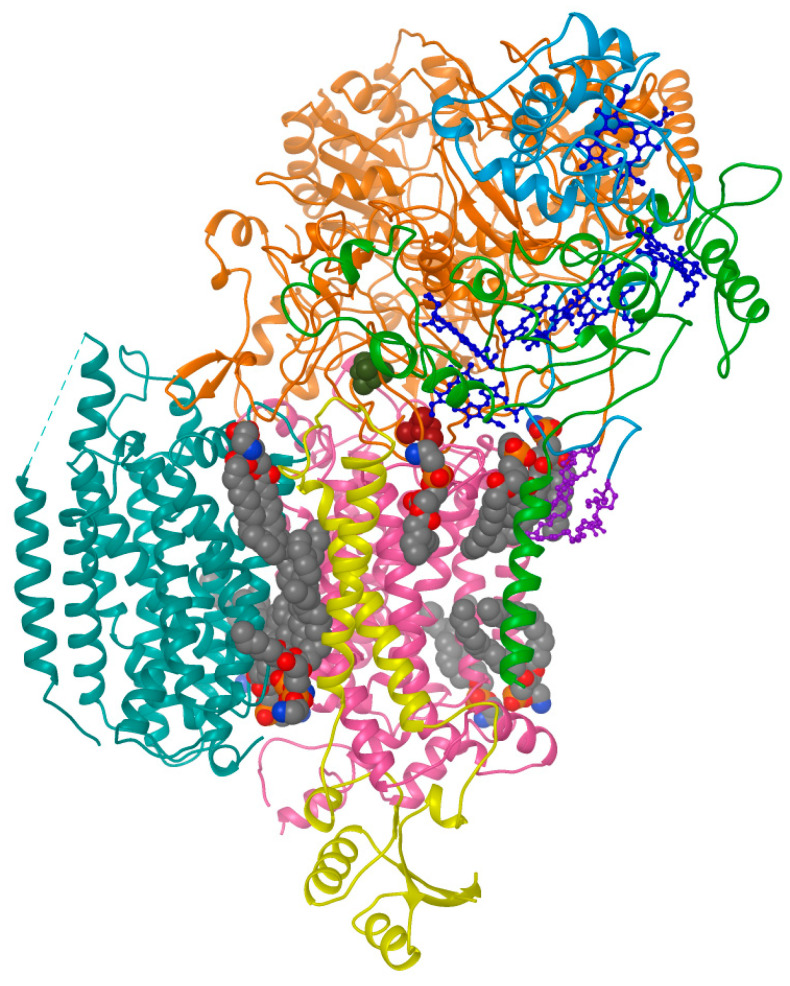
Structure of respiratory the Alternative Complex III (ACIII) and lipids within a SMALP, as determined by cryo-EM and deposited as EMD-7286 and EMD-7448 [55]. The ActA, ActB, ActC, ActD, ActE and ActF subunits are colored green, orange, red, yellow, light blue and cyan, while the haem, lipid anchors and phospholipid groups are dark blue, purple, and grey, respectively, as originally depicted in [69].

The confluence of native nanodisc and cryo-EM technologies has allowed determination of several memtein structures, revealing the positions of asymmetric bilayer leaflets and endogenous post-translational modifications in multisubunit complexes. The first memtein to be resolved was Alternative Complex III (ACIII), a 464 kDa complex from *Flavobacterium johnsoniae* that was solubilized using 1% SMA3000 as well as XIRAN 25010 copolymer [55], which is now formulated as SMALP 25010. One of the subunits binds to Ni-NTA metal affinity resin, allowing purification of the entire complex in SMALPs, which can adapt to accommodate the 9 × 13 nm assembly including six protein subunits with 48 transmembrane helices and an accessory cytochrome c oxidase. The structure was determined at 3.4 Å resolution and shows the orientations of acylated residues that carry out photosynthetic electron transport, as well as haem groups, iron–sulfur clusters and an array of phospholipid molecules (Figure 5).

The superfamily of ATP-binding cassette (ABC) transporters contains targets involved in cancer drug resistance and genetic diseases including adrenoleukodystrophy and cystic fibrosis. Five mammalian transporters were solubilized using SMA2000 (2.5% *w/v*), and were more pure and stable than with detergent preparations [64]. The human P-glycoprotein extracted into SMALPs from insect cells exhibits the expected interactions with doxorubicin substrate and verapamil inhibitor as well as monomer and dimer structures by cryo-EM. A subsequent study of the multidrug exporter AcrB from *E. coli* revealed the associated asymmetric bilayer at high resolution [53]. Following solubilization with SMA(2:1) copolymer and single-step purification using a His tag, the trimer could be resolved to 3.0 Å with a section of the co-purified *E. coli* membrane. A set of 31 lipid molecules form visible hexagonal patterns in the inner leaflet and ordered disarray in the outer leaflet. The first structure of the *Salmonella (S.) typhimurium* AcrB has been resolved at 4.6 Å following solubilization in SMA, allowing the conformational effects of disease-resistant mutation and dynamic changes to be modelled [54]. This provides avenues to deduce the mechanisms by which transporters bind and translocate specific lipids such as PIs [71] within transmembrane environments.

Ion channels form selective pores for ions to cross the membrane in response to changes in chemical stimulation, mechanical forces or temperature, and play roles in a wide range of physiological processes, including neuronal disorders. These multisubunit proteins respond to signaling lipids, which in some cases can regulate channel opening [72]. The zebrafish glycine receptor expressed as a homopentamer in insect cells can be solubilized with SMALP 30010 (0.5% *w/v*), and purified by metal affinity and size exclusion chromatography for cryo-EM analysis. Multiple structures of agonist-bound open, closed, and desensitized states were only achievable in SMALPs, with resolutions down to 2.9 Å. This allowed the positions of the bound glycine agonist, inhibitory γ-aminobutyric acid (GABA) neurotransmitter and taurine to be elucidated, with binding activities quantified with scintillation-proximity assay (SPA) [62]. The α1 subunit of the human glycine receptor expressed in *Xenopus* oocytes and HEK cells also forms a pentamer that can both be solubilized with SMA(3:1) copolymer (2% *w/v*). Comparison of the agonist binding responses indicates significant differences between the two preparations, suggesting that HEK cell lipid compositions are better suited for pore opening [61].

**Figure 6:**
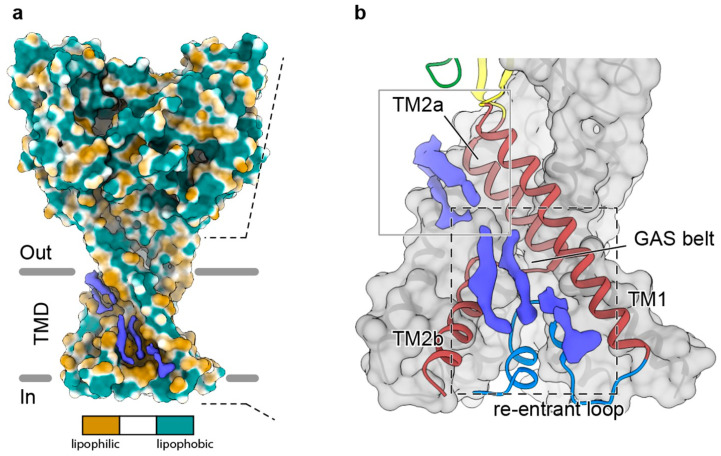
Structure of the ASIC1 ion channel in a SMALP and shown as a surface. (**a**) The exposed residues are colored based on whether they are lipophilic or lipophobic. (**b**) In the expansion the positions of lipid molecules are in blue along with the underlying transmembrane helices in red. The position of the “Gly-Ala-Ser” (GAS) belt element that forms a constriction within the channel is indicated. Taken from [57].

The acid-sensing ion channel ASIC1 is found in neurons and has been solubilized and resolved with bound lipids using SMALP 30010 copolymer. The structure of the trimeric channel was determined at pH 7 and 8 to resolutions of ~2.8 and 3.7 Å, respectively. These illuminate the long-lived desensitized and resting state conformations, which are distinguished by the cavities and re-entrant loop positions within the channel. Several lipid densities are also uniquely evident in the SMALP preparations, indicating the positions of biological-source lipids along the transmembrane helices (Figure 6). The *Bacillus subtilis* KimA protein is a K^+^/H^+^ symporter that has been solubilized with SMALP 30010 [63]. This allowed its homodimeric structure to be resolved by cryo-EM to 3.7 Å, including positions of 24 transmembrane helices and three potassium ions within the channel. The exchange of CO_2_ and water vapor in plants is mediated by the slow anion channel 1 (SLAC1). The structure of this channel from a brome grass has been resolved at 2.97 Å after expression in *Schizosaccharomyces (S.) pombe* and solubilization with SMA copolymer [67]. The positions of several lipid molecules can be discerned at the center and periphery of the symmetric trimer at positions expected within the bilayer.

Lipids including PIs are also known to modulate activities of various channels based on patch-clamp studies [73]. Most structures reveal how components can be assembled from synthetic and recombinant sources. A set of cryo-EM structures of two-pore channels bound to exogenously added short-chain PI(3,5)P_2_ lipids indicate effects on pore conformations and specific activation [74,75], and suggest that native nanodiscs could be used to illuminate their mechanisms in bilayers. The interactions of dioctanoyl PI(4,5)P_2_ and calmodulin with the Ca^2+^ selective channel TRPV5 in its open state has also been resolved by cryo-EM, revealing structural rearrangements around the gate [76]. The closed state of the TRPM8 channel bound to the same short-chain PI(4,5)P_2_ ligand reveals that this lipid occupies a distinct interfacial cavity, indicating variable mechanisms for lipid-activation in this family [77]. Crystal structures of classical inward rectifier (Kir2) and G protein-gated K^+^ channel bound to PI(4,5)P_2_ show how this lipid induces conformational changes and influences channel gate opening [78,79]. High resolution structures showing how long-chain PIs or bilayers engage in vivo remain elusive, although natural extracts can be added. For example, a phosphatidylinositol molecule from soybean lipid extract is seen to occlude the agonist-binding pocket of the closed TRPV1 channel in MSP nanodiscs, and would presumably be expelled upon agonist binding or elevated temperature [80].

The structure of the mechanosensitive channel YnaI has been determined in several states following expression in *E. coli* and solubilization with DIBMA copolymer. The heptamer was resolved by cryo-EM to 3.0 Å, displaying a bilayer of lipid molecules including a contact with a key Lys residue [14]. The formation of open-like and intermediate states from the closed form can be induced by incorporation of lysoPC into the discs. Four conformers are apparent and exhibit various transmembrane helix positions, bend angles and pore size, indicating how bilayer deformation transduces forces across the membrane. A variety of other lipids, most notably PIs, bind to and stabilize some mechanosensitive channels, [81], suggesting that they may also play roles in transduction that merit further investigation.

## 5. Recapitulating Function in Polymer-Stabilized Memteins as Exemplified by A_2A_R

The adenosine A2A receptor (A_2A_R) is a prototypical and ubiquitous class A receptor, of interest in the pharmacological treatment of specific cardiovascular disorders, immune response and wound repair, cancer, and central nervous system disorders [82,83,84,85]. While specific detergent mixtures, in combination with fusion constructs or nanobodies [86,87,88,89], can be used to obtain both inactive- and active-state signatures of A_2A_R, ligand affinities are typically an order of magnitude lower in detergents than in liposomes or cell membranes. This implies that the cell or membrane environment plays a significant role in modulating receptor function. As GPCR reconstitution efforts have improved, it has become possible to directly interrogate the stabilizing effects of phospholipid mixtures over detergent [31,90,91]. Of note, agonist affinities and receptor stability increase precipitously in phospholipid. At the same time, G protein coupling and ligand-dependent modulation of G protein action, as measured through nucleotide exchange or GTP hydrolysis assays, is rarely possible without resorting to reconstituted phospholipid bilayer assemblies [91]. In contrast, while some of the earlier ligand binding assays in SMALPs clearly demonstrated that a GPCR could be reconstituted [36], activation and G protein coupling is much more stringent and is expected to require an appropriate lipid interface in addition to an electrostatic surface potential that allows binding an activation of the G protein. The interaction of the cytosolic interface of A_2A_R with the alpha-subunit of the G protein is largely driven by electrostatic interactions which would likely be screened by the acidic nature of early-generation polymeric scaffolds.

**Figure 7:**
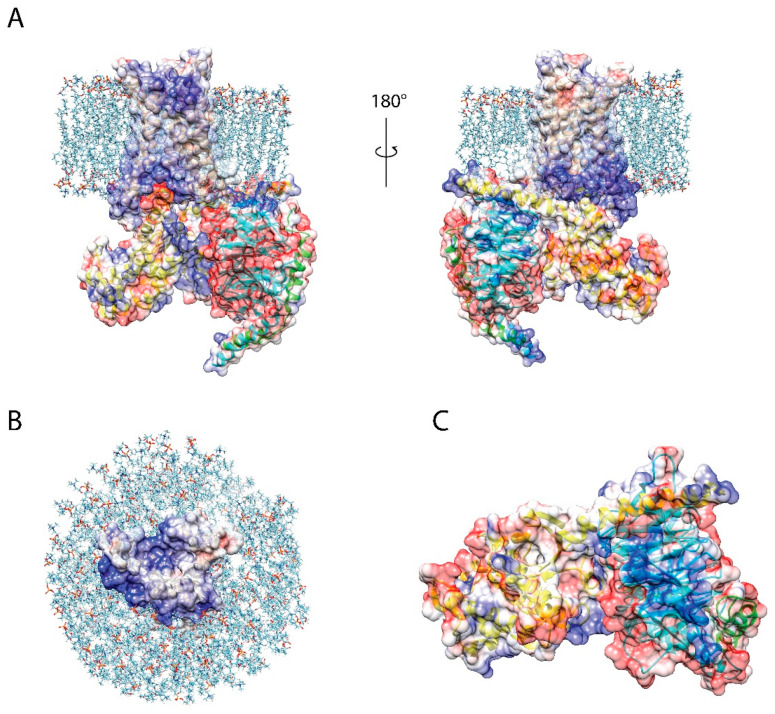
Cartoon representation of the A_2A_R–G protein complex, in a lipid bilayer, showing the importance of electrostatic surface for complex formation. (**A**) Electrostatic surface at the receptor–G protein interface. (**B**) Electrostatic surface of the intracellular domain of A_2A_R. (**C**) Electrostatic map of the interaction surface of Gs.

A_2A_R function is also likely modulated by specific lipids and physiological adjuvants. In a recent molecular dynamics simulation study, the authors investigated preferential interactions between a variety of GPCRs and various charged lipids, cholesterol, fatty acids, saturated and unsaturated phospholipids [92]. Cholesterol, and charged lipids in particular, are often found to interact with specific motifs in many receptors. In the case of A_2A_R, specific cholesterol interaction motifs have been proposed to play an allosteric role in enhancing receptor signaling [93,94]. More recently, PI(4,5)P_2_ was identified as a key allosteric mediator in the A_2A_R–G protein complex [24]. Sodium is also well known to bind to a conserved motif in the majority of class A GPCRs and likely plays a key regulatory role in modulating receptor activation, as do magnesium and calcium ions [95]. Thus, as suggested in Figure 7, the need to avoid destabilizing electrostatic interference, the preservation of lipid composition and transmembrane heterogeneity, and the capacity to tolerate the presence of cationic salts are quite probably essential criteria in polymer design to recapitulate physiological receptor function.

## 6. Polymer Design for Native Nanodiscs

The discovery that SMA(2:1) copolymer could be used to rapidly convert membranes into native nanodiscs (SMALPs) for structure–function analysis [4] has heralded the advent of ex vivo structural biology. Remarkably, 12 years later, this polymer remains the most efficient tool for extraction of most memteins from diverse biological sources. Standard protocols for the preparation and use of polymers have been developed [96,97] and ready to use solutions and kits are now available from Cube Biotech and Orbiscope. A native cell membrane nanoparticle (NCMN) system has also been developed to optimize extraction and purification using a variety of copolymers including a taurine derivative [9]. Ongoing efforts are focussing on improving utility for structural and functional analysis of diverse targets, particularly those that are labile or of low abundance, and a reduction in any complicating issues such as precipitation that can occur with high divalent cation levels or low pH, leading to a generation of new polymers (Table 2).

Grafting of different polar sidechains can enhance polymer solubility and may offer handles for visualization and purification (Figure 1, Table 1). The derivatization of SMA with sidechains such as ethanolamine (SMA-EA) or ethylenediamine (SMA-ED) expands the accessible pH range and tolerance of high divalent cation levels [6,7]. Conversion of SMA-ED to its dehydrated SMAd-A form allows solubilization of membranes below pH 6. The grafting of tertiary and quaternary ammonium cations to SMA to yields SMI and SMA-QA, respectively, which are compatible with broad pH and divalent cation ranges [10,11]. Although conventional SMALPs can adapt to the size and shape of their cargo, larger diameter nanodiscs can also be produced using copolymers such as zwitterionic SMA (zSMA), which bears phosphatidylcholine groups [15]. Larger discs that gently solubilize multimers and fibrils can also be produced using methylamine (SMA-MA), ethylamine (SMA-EtA), and propylamine (SMA-PA) copolymer derivatives, which solubilize memteins over a wide pH range [16,17].

Another goal has been the unambiguous detection of copolymer concentrations in a manner that does not overlap other signals of protein cargo. A variety of fluorescent or affinity tags can be attached to a thiol-containing version such as SMA-SH in order to detect and purify polymers and native nanodiscs [8]. In DIBMA copolymers the styrene group is replaced with aliphatic moieties that are better suited to circular dichroism (CD) analysis of embedded proteins. These are also compatible with higher levels of divalent cations than conventional SMA [17], and have been used to solubilize channels [68] and GPCRs [98]. This backbone can also be derivatized with glucosamine and glycerol sidechains to expand solubility and utility. A series of PMA copolymers with various ratios of butyl and acetylcholine sidechains fragment membranes into particles that are useful for CD studies [16]. A set APAA copolymers which are based on alkyl (butyl, pentyl, or hexyl) polyacrylic acid convert membranes into nanodiscs with diameters between 7 and 17 nm, with longer sidechains engendering greater efficiency [15].

The need to enhance resolution has led to efforts to reduce the dispersity in comonomer sequence distribution, composition and chain length (Ð) of copolymers. Comparative studies of compositionally defined SMA copolymers shows that the kinetics of lipid solubilization kinetics is dependent on the length of copolymer chains, with shorter copolymers solubilizing membranes faster than longer ones [99]. Acrylic acid and styrene (AASTY) copolymers exhibiting reduced Ð have been used to characterize the structure of the transient receptor channel TRPM4, albeit at low resolution [13].

The use of methyl-substituted stilbenes in copolymerization reactions (rather than styrene) along with maleic anhydride generates STMA copolymers that possess semi-rigid backbones [100,101,102]. The unique composition, sequence distribution, and backbone rigidity of these copolymers confer broader pH utility and more homogeneous nanodisc sizes and shapes, as demonstrated with the *E. coli* membrane protein PagP [12]. Further development of such copolymers will enable a continued growth of this field as a wider diversity of membranes, tissues and targets can be exploited for an expanding range of applications.

## 7. Conclusions

A significant body of evidence of how native nanodiscs can be produced by a family of SMA-related polymers has emerged over the past decade. This is allowing researchers to make new inroads into the structural biology and drug discovery applications of native membrane assemblies. The range of polymer types is allowing structure–activity relationships to emerge, informing the development of new polymers that are zwitterionic, tagged, more homogeneous, conformationally restrained and/or circularized for giving optimal performance for demanding transmembrane targets. Such tools are allowing the preparation of endogenous complexes that are unsuitable for detergent extraction or reassembly for structure–function analysis and high-throughput screening. Challenges remain including the isolation of in vivo states of mammalian protein multimers that are poorly expressed or metastable as well as the transient complexes with signaling partners. Some membrane assemblies present technical issues due to reactivity or fragility and still require careful optimization in order to obtain sufficient material for biophysical analysis, and some polymers may influence activities of proteins and affinity tags due to spurious interactions. While the polydispersity of synthetic polymers may be useful for solubilizing the mixed populations of memteins found in cells, this property can limit the resolution of structural data. The sizes of discs may be limiting in the cases of very large targets, although they generally appear to be able to adapt to wrap variously shaped protein cargo bound to a bilayer of lipid. Polymers with integrated tags are being designed for much more convenient purification and visualization of membrane proteins with minimal impact of their function. Such tools could prove useful in screening campaigns for small molecule and antibody ligands, and may unlock a wide array of applications in fields ranging from proteomics, lipidomics and metabolomics. The recent effort of the SMALP community to design improved polymers to enable memtein structure determination is geared to support the discovery of therapeutic agents for native-state targets as well as ongoing advances in fundamental research into membrane biology.

## Figures and Tables

**Table 1:** Structures and interactions of selected transmembrane targets solubilized in native nanodiscs. The proteins including acriflavin resistance protein AcrB and transient receptor potential cation channel subfamily M member 4 are listed along with their superfamilies, bound lipids and ligands, molecular weight (MWt) in kilodaltons, expression hosts, type of structural analysis, references and resolutions unless not determined (nd).

Protein	Family	MW (kDa)	Subunits	Tag	Host	Copolymer	Assays	Lipids	Ligands	Resol.	Refs.
A2AR	GPCR	45	1	Nt-His_10_	*P. pastoris,* HEK 293T	SMA2000, SMI	AUC, CD, ^3^H-ligand binding, fluorescence spec.	PE, PC	ZM241385, XAC, NECA, theophylline	-	[10,36,37,38]
AcrB (*E. coli*)	transporter	344	3	Ct-His	*E. coli*	SMA2000	cryo-EM	PE	-	3.0 Å	[53]
AcrB (*S. typhimurium*)	transporter	341	3	Ct-His	*E. coli*	SMA	cryo-EM	nd	-	4.6 Å	[54]
Act-A, -B, -C, -D, -E, -F	photosystem	464	6		*F. johnsoniae*	SMA3000, SMALP 25010	cryo-EM	PE	heme, cyt aa3	3.4 Å	[55]
AR3	7TM	27	3	-	*H. sodomense*	SMA3000	CD, DLS, MS/MS	DMPC, S-DGD, 2DP	retinal	-	[56]
ASIC1a (*Gallus gallus*)	ion channel	180	3	His-eGFP	HEK293S	SMALP 30010	cryo-EM	nd	^1^H	2.8 Å	[57]
bacteriorhodopsin	7TM	27	1	-	*H. salinarum*	SMA3000	CD, DLS, MS/MS	DMPC, 2DP	retinal	-	[56]
bacteriorhodopsin (*R. xylanophilus*)	7TM	310	12	Ct-His_6_	*E. coli*	SMA2000	AFM, DLS	*E. coli*	retinal	-	[58]
bacteriorhodopsin (*H. w.*)	7TM	88	3	Ct-(His_6_)_2_	*E. coli*	SMALP 25010	LCP-XRD	monoolein	retinal	2.0 Å	[59]
bacteriorhodopsin (*N. pharaonis*)	7TM	76	3	Ct-His_6_	*E. coli*	SMA3000	EPR	*E. coli*	retinal	-	[60]
bacteriorhodopsin (*N. pharaonis*)	7TM	76	3	Ct-His_6_	*E. coli*	DIBMA	AFM, DLS, EPR, TEM	*E. coli*	retinal	-	[18,60]
β2-adrenoceptor (β2AR)	GPCR	75	1	Nt-Strep, SNAP	HEK	DIBMA	FRET, PTS	nd	propranolol, isoprenaline	-	[52]
Cannabinoid receptor 1 (CB1)	GPCR	53	1	Nt-FLAG, Ct-GFP, His_8_	Sf9	SMA2000	DLS, SPR, flow cytometry	nd	antibody	-	[43]
Dopamine D1 receptor (D1R)	GPCR	49	2	Nt-His_6_	HEK293f	SMA3000	^3^H, MST-ligand binding, CD	nd	neurotensin peptide, SCH23390	-	[40]
D1R:GHSR	GPCR	181	4	Nt-(His_6_)_2_	*P. pastoris*	SMA(2:1)	ligand binding	nd	Gα_q_, G_βγ_	-	[41]
GHSR1a	GPCR	41	1	His	*E. coli*	SMA2000, SMA3000	Fluorescence emission	-	Ghrelin, SPA	-	[42]
GlyR α1 (*H. sapiens*)	ion channel	240	5	Ct-His_6_	*Xenopus* oocytes, HEK293	SMA3000	MST	nd	glycine, taurine	-	[61]
GlyR α1 (*D. rerio*)	ion channel	253	5	Ct-His_8_	Sf9	SMALP 30010	cryo-EM, SPA	nd	GABA, glycine, taurine	2.9 Å	[62]
KimA	transporter	136	2	Ct-His_10_	*E. coli*	SMALP 30010	cryo-EM	nd	K^+^ (3)	3.7 Å	[63]
MT1R	GPCR	40	1	His	CHO-K1, *P. pastoris*	SMA2000, SMA3000	Fluorescence, ligand binding	-	Gα_q_β_1_γ_2,_ β-arrestin	-	[42]
NTSR1	GPCR	42.5	1	Nt-FLAG, Ct-eGFP-His_10_	Sf9	PMA	GTPase-Glo	nd	G_i1_ and G_q_ heterotrimers	-	[50]
P-glycoprotein (ABCB1)	transporter	141	1,2	Ct-His_12_	Sf9	SMA2000	AUC, CD, cryo-EM	nd	ATP, verapamil, doxorubicin	35 Å	[64]
Prion	prion	28	fibril	-	brain	SMA-MA	TLC, TEM	PE, PC	-	-	[65]
PTH1R	GPCR	53			HEK293	SMA2000	HDX-MS	nd	antibody	-	[66]
SLAC1	ion channel	190	3	Ct-Flag, His_10_	*S. pombe*	SMA	Cryo-EM, MS/MS,	sphingo- lipid	-	3.0 Å	[67]
TRPM4	ion channel	537	4		HEK293	AASTY	cryo-EM	nd	-	18 Å	[13]
V_1a_R	GPCR	40		na	HEK 293T	SMI	Ligand binding	nd	vasopressin	-	[10]
YnaI channel	ion channel	40			*E. coli*	DIBMA	cryo-EM	PE/PC	LPC	3.0 Å	[68]

**Table 2:** Comonomer ratios, molecular weights and polydispersities of SMA-derivative and SMA-like copolymers used to solubilize membranes into native nanodiscs as well as the relevant references.

Copolymer	Apolar Subunit	Polar Subunit	Subunit Ratio	M_n_^a^ (g/mol)	Ð^a^	References
2-STMA	stilbene	maleic acid	1:1	4400	1.19	[12]
4-STMA	stilbene	maleic acid	1:1	5800	1.54	[12]
AASTY	styrene	acrylic acid	1:1	8900		[13]
APAA	alkyl	acrylic acid				[15]
DIBMA	alkyl	maleic acid	1:1	8500	1.4	[17]
DIBMA glucosamine	alkyl	maleamic acid-glucose	1:1			Cube Biotech (CB)
DIBMA glycerol	alkyl	maleamic acid-propanediol	1:1			CB
PMA	butyl acrylate	acetylcholine	1:1.1	6900		[16]
SMA2000	styrene	maleic acid	2:1	3000	2.5	[4]
SMA3000	styrene	maleic acid	3:1	3800	2.5	[4]
SMA-EA	styrene	maleamic acid-ethanolamine	1.3:1	1600		[6]
SMA-ED	styrene	maleamic acid-ethylene-diamine	1.3:1	1600		[7]
SMA-EtA	styrene	maleamic acid-ethylamine	1:1			[5]
SMA-MA	styrene	maleamic acid-methylamine	1:1			[5]
SMA-PA	styrene	maleamic acid-propylamine	1:1			[5]
SMA-QA	styrene	maleimide-ethyl-trimethylammonium	1.3:1	1600		[11]
SMA-SH	styrene	maleamic acid-cysteamine	2:1			[8]
SMA-Tau/NCMNP7	styrene	maleamic acid-taurine	2:1		2.5	[9]
SMAd-A	styrene	maleimide-ethanolamine	1.3:1	1600		[7]
SMALP 1100I	styrene	maleimide-propyl-dimethylamine	1.4:1	5000	2.5	CB, Orbiscope
SMALP 25010	styrene	maleic acid	3:1	4000	2.5	CB, Orbiscope, [4]
SMALP 30010	styrene	maleic acid	2.3:1	2500	2.6	CB, Orbiscope, [4]
SMALP 40005	styrene	maleic acid	1.2:1	2000	2.5	CB, Orbiscope
SMALP 502-E	styrene	esterified maleic acid	1.5:1			CB, Orbiscope
SMI	styrene	maleimide-propyl-dimethylamine	2:1	2700	2.8	[10]
zSMA1	styrene	phosphatidylcholine			1.1	[14]
zSMA2	styrene	phosphatidylcholine		35,000	1.17	[14]
zSMA3	styrene	phosphatidylcholine		53,000	1.19	[14]

## Data Availability

The data used here are publicly available.
